# Spectral measures and mixed models as valuable tools for investigating controls on land surface phenology in high arctic Greenland

**DOI:** 10.1186/1472-6785-7-9

**Published:** 2007-09-19

**Authors:** Mikkel P Tamstorf, Lotte Illeris, Birger U Hansen, Mary Wisz

**Affiliations:** 1 University of Aarhus, National Environmental Research Institute, Dep. for Arctic Environment, Frederiksborgvej 399, PO Box 358, DK-4000 Roskilde, Denmark; 2 University of Copenhagen, Institute of Biology, Øster Farimagsgade 2D, DK-1353 Copenhagen K, Denmark; 3 University of Copenhagen, Department of Geography and Geology, Øster Voldgade 10, DK-1354 Copenhagen C, Denmark

## Abstract

**Background:**

Changes in land surface phenology are of major importance to the understanding of the impact of recent and future climate changes in the Arctic. This paper presents an extensive study from Zackenberg Ecological Research Operations (ZERO) where snow melt, climate and growing season characteristics of six major high arctic vegetation types has been monitored during 1999 to 2005. We investigate the growth dynamics for dry, mesic and wet types using hand held measurements of far red normalised difference vegetation index (NDVI-FR) and generalized additive mixed models (GAMM).

**Results:**

Snow melt and temperature are of major importance for the timing of the maximum growth as well as for the seasonal growth. More than 85% of the variance in timing of the maximum growth is explained by the models and similar for the seasonal growth of mesic and wet vegetation types. We find several non-linear growth responses to the environmental variables.

**Conclusion:**

We conclude that the uses of GAMMs are valuable for investigating growth dynamics in the Arctic. Contrary to several other studies in the Arctic we found a significant decreasing trend of the seasonally integrated NDVI-FR (SINDVI) in some vegetation types. This indicates that although greening might occur wide-spread in the Arctic there are variations on the local scale that might influence the regional trends on the longer term.

## Background

Land surface phenology is a key variables for modelling of the terrestrial ecosystems in a global change perspective and as such as input into circulation models (GCM's) [1]. Recent models agree that changes in vegetation and soil processes will have net positive feedback on future global warming [2]. Further, the way terrestrial ecology is implemented in GCM's will have a strong impact on the ability to predict future climatic changes [3] and knowledge of changes in the vegetation cover and the reasons for these are therefore of major importance. This has led to a number of publications and assessments on the greening and impact of global climate change in northern high latitudes [4-6] based on monitoring studies [7] and experimental studies [8,9]. Several of these studies have investigated the effect of temperature, light and fertilization on photosynthesis [10,11] and found that fertilization and increased temperatures significantly increases the photosynthesis while shading decreases the photosynthesis. Thawing degree-days and time of snow cover melt was found as the dominating controls on the phenology in the Subarctic [12]. Recently, Walker et al. included warming experiments from 11 locations across the tundra biome in a meta-analysis of the response of tundra vegetation to predicted warming [13]. They found a clear response where warming increased height and cover of dwarf shrubs while moss, lichen cover and species diversity decreased. However, the high arctic sites showed a lack of response believed to be based on the limited ability for the high arctic plants to respond to changes in the growing season temperature and length. Very few studies of the controls on arctic vegetation phenology have been performed in the High Arctic. The most extensive study from high arctic Greenland dates back to the 1930'ies where Sørensen found that temperature and timing of snow melt were the most important factors for phenological responses [14]. More recently, Marchand et al. used experimental warming on high arctic grasslands, finding that warming enhanced the green cover [15]. A clear control of temperature and photosynthetically active radiation (PAR) on the timing of flowering was found by Høye et al. who investigated the phenology and flowering of Dryas spp. hybrids along a snowmelt gradient in Northeast Greenland [16].

Normalised Difference Vegetation Index (NDVI) is a spectral measure that has been used widely in some of these studies as proxy for biophysical variables like biomass, leaf area index, CO2 flux etc. and that can be acquired non-destructively. Vegetation reflects light differently in the red and infrared wavelength resulting in a measure that is highly correlated with the biophysical variables [17]. This enables long-term monitoring of land surface phenology using the same plots instead of traditional destructive methods where plant material is harvested for weighing, leaf area measurements etc. Several authors have tested different phenological models for use with NDVI and land surface phenology including double-logistic functions [18], sigmoid functions [19], quadratic functions [20] and others. However, Hope et al. used the low-resolution AVHRR satellites to investigate trends and possible controls of NDVI on the North Slope of Alaska (1989–1996) [21]. They focussed on the seasonal integrated NDVI (SINDVI) over three different vegetation types and found no relation between meteorological observations and SINDVI using standard correlations. They speculated that the complex interactions between climate and vegetation might require more complex modelling to reveal the relationships between these factors. Karlsen et al. used a similar integrated measure (TI NDVI) for the study of inter-annual changes in land surface phenology [22].

**Figure 1:**
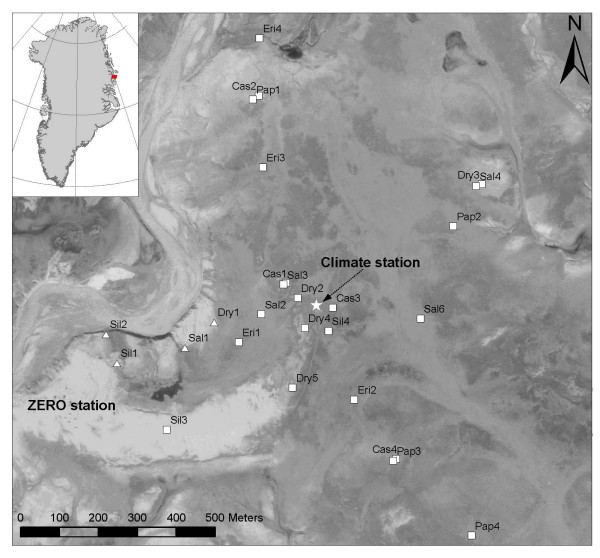
**Zackenberg valley area and location of NDVI-FR plots**. Location of the Zackenberg Ecological Research Operations (ZERO) station and the climate station (star) is shown. The NDVI-FR plots shown as triangles have not been used in this study due to very early snow melt (see text). NDVI-FR plots (naming, area etc) are described in detail in Meltofte and Berg (2004). Background image is a grey-scale ortho photo from 7 August 2000.

In this paper we present a seven year field study of land surface phenology in the High Arctic and use mixed models to investigate the possible controls of the growth dynamics. The NDVI-FR measurements are obtained from 26 individual plots within the Zackenberg Ecological Research Operations (ZERO) area (Figure 1) using a hand held Skye Instruments sensor at six vegetation types that range over dry, mesic and wet types. This sensor uses the red and far red bands instead of the traditional red and near-infrared bands. We investigate the seasonally integrated far red normalised difference vegetation index (NDVI-FR) using the nomenclature of Hope et al. (SINDVI) [21], the timing of the maximum NDVI-FR (DOYmax) and the level of maximum NDVI-FR (MaxNDVI). We hypothesize that the main explanatory variables controlling the seasonal growth dynamics of the six main vegetation types are timing of the snow melt, temperature during the growing season, light in the form of photosynthetic active radiation, rain during the growing season, the general state of the vegetation as expressed by the SINDVI in the previous growing season and the temperature during the previous year growing season. We expect that the different vegetation types will show different response from the explanatory variables.

## Results

### NDVI-FR seasonal modelling

**Figure 2:**
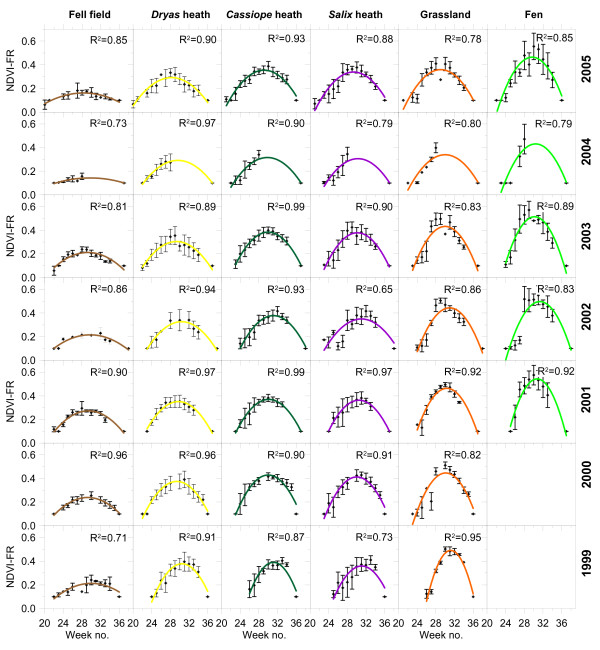
**Summary of all NDVI-FR measurements**. Error bars indicate one standard deviation.

The difference in seasonal greenness and onset of growing season between vegetation types is clear in Figure 2. It shows a summary of all the used NDVI-FR data for the six vegetation types during 1999 to 2005. In the figure the quadratic model has been fitted to the average NDVI-FR of each vegetation type for each week. For the 783 separate dataset (Table 1) the quadratic models fitted with a mean R2 value of 0.92. The dry and sparsely vegetated fell field melts free from snow earlier than the rest of the vegetation types but are more sparsely vegetated and therefore less green. From the relative dry dwarf shrub heaths (Dryas and Cassiope) over the moist Salix heath to the more wet grassland and fens later snow melt and higher maximum NDVI-FR values are clear. Looking across years, 1999 was a record year with deep snow cover and low spring temperatures causing the growing season to start very late. Contrary, 2005 had low snow cover and high temperatures both through winter (several thaw events) and during spring causing an early start of the growing season.

**Table 1:** Number of observations for the environmental variables used in the modelling

	2000	2001	2002	2003	2004	2005	All years
Fell field	10	10	9	9	9	9	56
*Dryas heath*	23	23	23	23	23	23	138
*Cassiope heath*	45	45	45	45	45	45	270
*Salix *heath	25	25	25	25	23	30	153
Grassland	9	9	9	9	9	9	54
Fen	0	0	32	32	24	24	112

All types	112	112	143	143	133	140	783

### Interannual and seasonal characteristics of the vegetation types

**Figure 3:**
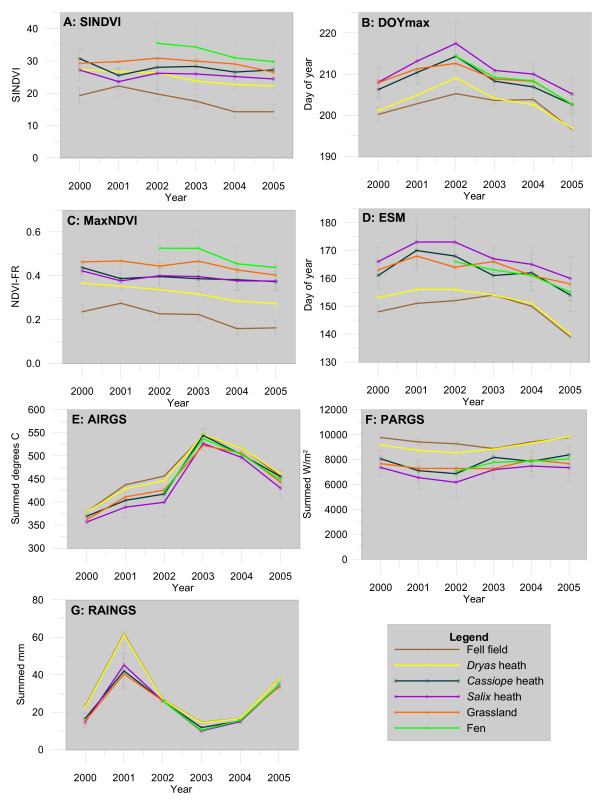
**Mean response variable and variable data for each vegetation type and year**. SINDVI: Seasonal Integrated NDVI-FR, ESM: End of snow melt, DOYmax: Day of maximum growth, AIRGS: summed air temperatures above 0 degrees during the growing season, MaxNDVI: Maximum occurring NDVI-FR, PARGS: summed photosynthetic active radiation during the growing season, RAINGS: summed rain during the growing season. Bars indicate one standard deviation.

Summary of the major growth dynamics variables (SINDVI, DOYmax and MaxNDVI) and exploratory variables (snow melt, temperature, light and rain) for each vegetation type and each year is shown in Figure 3. Only the full season data are shown. 1999 is only used as the previous year data in 2000 and therefore not shown directly. The data show that the 6 vegetation types have different growth dynamics and grow under different abiotic conditions. Especially the dry types (fell field and Dryas heath) show different characteristics than the other types. They experience an earlier snowmelt and therefore earlier start on the growing season. This results in a higher input of photosynthetical radiation partly since the radiation is higher early than later during the summer and partly due to the longer growing season. Also the summed temperatures are slightly higher for the dry types than for the other types. Fell field and Dryas heath have an earlier maximum of the growth season but a lower maximum NDVI-FR and lower seasonal integrated NDVI-FR than the rest. Salix heath is the vegetation type that melts out latest in the season and peaks later than the other types. Contrary to the dry types it has a shorter growing season and hence experience lower summed temperatures and PAR (Photosynthetic Active Radiation). Salix heath has a medium MaxNDVI that does not change much between years. The highest NDVI-FR values occur in the more wet vegetation types, grassland and fen. Fen has the highest maximum values and highest SINDVI but experiences abiotic conditions close to average for the area. Most of the vegetation types show a significant trend towards earlier snow melt and lower MaxNDVI. Changes between years in MaxNDVI are small.

Before running the models we tested for correlation between each set of explanatory variable. We did not find any correlations with R2 higher than 0.6 among the used variables and therefore included all in the modelling. High correlation is on the other hand found between two of the main variables, MaxNDVI and SINDVI with distinct grouping of the vegetation types along the possible regression line – dry types at the bottom and more moist and vigorous types at the top rigth. The same pattern was reported by Hope et al. [21].

### GAMM modelling

The modelling of SINDVI and DOYmax was completed with success for each of the vegetation types and for the entire datasets. However, the modelling of MaxNDVI was not successful. The models did not converge with 3 degrees of smoothing and when we tried to raise the numbers of smoothing we found that it resulted in unreasonable responses that had no biological meaning. Table 2 shows a summary of the results of the modelling of SINDVI and DOYmax. For each response variable is shown the included explanatory variables of the seven models and an indication for the direction of the correlation of each variable. The total variance that was explained is shown for each model as the adjusted R2.

**Table 2:** Final model results

**SINDVI**	All	Fell field	*Dryas*	*Cassiope*	*Salix *heath	Grassland	Fen
Time of snow-melt	(-)		∩	(-)	∩	(+)	+
Temperature (during growing season)	∩	+	(-)	∩	∩	∩	
Light (during growing season)	(-)	+		(-)	U		(+)
Rain (during growing season)	(-)	+		(-)	(-)	(-)	+
SINDVI, prev. year	+	+	(+)	(+)	(+)		(+)
Temperature, prev. year	(-)	-	-	-	-	-	-

Variance explained by model (adj. R^2^)	0.78	0.84	0.21	0.55	0.79	0.53	0.82

**DOYMAX**	All	Fell field	*Dryas*	*Cassiope*	*Salix *heath	Grassland	Fen

Time of snow-melt	(+)	(+)	(+)	(+)	(+)	U	+
Temperature (during green-up)	(+)		(+)	(+)	(+)	U	U
Light (during green-up*)		+		(+)	(+)		
Rain (during green-up*)	∩	∩	U	(+)	(+)	∩	∩
SINDVI, prev. year				-		(+)	U
Temperature, prev. year	∩	(+)	-	∩	∩	∩	U

Variance explained by model (adj. R^2^)	0.89	0.98	0.85	0.97	0.98	0.89	0.98

Variables included in the final models and their relation to the modeled response variables: seasonal integrated NDVI-FR (SINDVI) and the timing of maximum NDVI-FR (DOYMAX). '+' indicates that the variable has a linear positive correlation with the response variable while '-' indicates a negative linear relation. Brackets indicate a non-linear correlation but with an overall positive or negative trend. '∩' indicate a non-linear response with a maximum, and 'U' indicate a non-linear response with a minimum. Blank indicates that the variable didn't improve the model and was therefore not included * Green-up is defined from the start of the growing season to the day of maximum.

**Figure 4:**
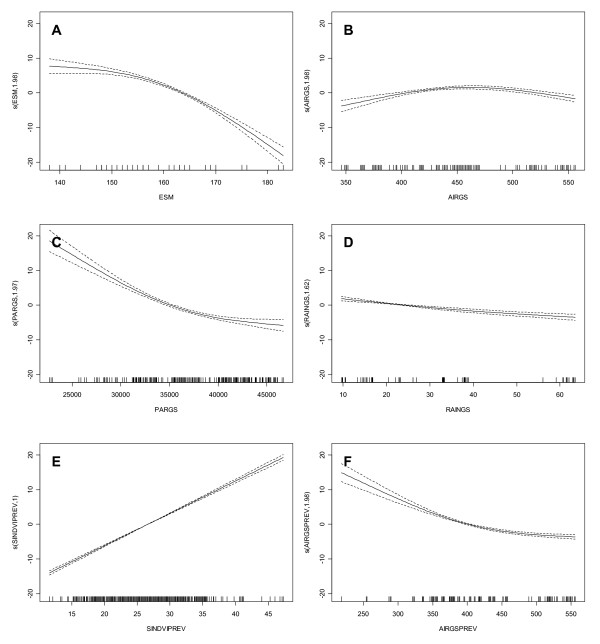
**SINDVI model results with all vegetation types included**. Each graph shows the response curve between each explanatory variable and SINDVI. Each response curve is the result of backfitting the GAMM model to calculate the additive contribution of each variable using non parametric smoothing methods. Thus, the y-axis can be interpretted as a transformation of SINDVI. Low values on the y-axis correlate with low SINDVI, while high values correlate with higher SINDVI. Dashed lines indicate twice standard errors. Each short bar on the x-axis indicates an observation. A: End of snow melt, B: summed air temperature during the growing season, C: summed PAR during the growing season, D: summed rain during the growing season, E: seasonal integrated NDVI-FR in the previous growing season, F: summed air temperature during the previous growing season. Estimated degrees of freedom are shown by each Y-axis.

SINDVI GAMM models: The SINDVI model for the overall vegetation cover (all types included in the model) show that all the variables hypothesized to control the SINDVI was significant and therefore included (Figure 4 and Table 2). 78% of the variance in SINDVI is explained by this model. SINDVI decreases non-linearly with late snow melt so that early snow melt before DOY 153 (1 June) does not seem to change SINDVI much but after this time SINDVI decreases sharply with later snow melt. The opposite is true for the amount of light (high summed PAR) and temperatures in the previous year where changes in the lower range has higher relative impact than with much light and high temperatures in the previous year. The relationship between rain and SINDVI is almost linearly in the observed range so that more rain during the summer will decrease the SINDVI. However, the relationship is not as pronounced as with the other variables. SINDVI increases linearly with the integrated vegetation greenness in the previous year (SINDVIPREV) as would be expected while it seems that the current year's air temperature is beneficial to SINDVI only until a curtain limit where after it influences negatively.

This behaviour of SINDVI to the explanatory variables is to some degree different when looking at the individual vegetation types. Only the negative effect of air temperature in the previous year on SINDVI is the same for all vegetation types. The fell field model did not include the time of snow melt in the model and had as opposed to the overall model a positive, linear relation between temperature, light, rain and previous year's SINDVI. With 84% of the variance explained fell field is the vegetation type with the most variance in SINDVI explained by the model. Cassiope heath show the same results as for the combined vegetation types but the Dryas heath model did not include light and rain in the model and for snow melt the response in SINDVI is positive with later melt until a maximum in the beginning of June (DOY 153) where SINDVI thereafter respond negatively to later snow melt. Only 21% of the variance in SINDVI is explained by the model. The pattern changes more going towards the more wet vegetation types until fen where SINDVI (like fell field) respond positive to later snow melt, increasing light and added rain.

**Figure 5:**
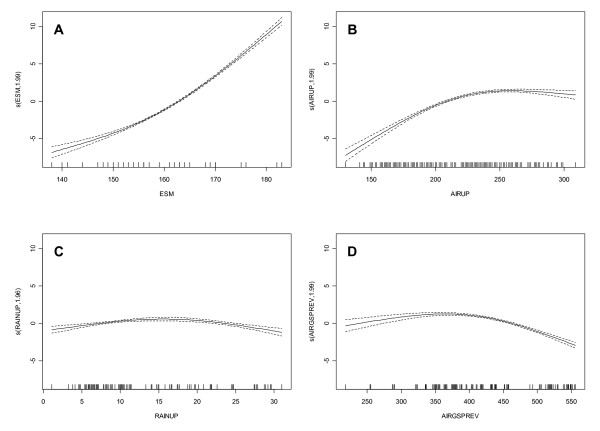
**DOYmax model results with all vegetation types included**. Each graph shows the response curve between each explanatory variable and DOYmax. Each response curve is the result of backfitting the GAMM model to calculate the additive contribution of each variable using non parametric smoothing methods. Thus, the y-axis can be interpretted as a transformation of DOYmax. Low values on the y-axis correlate with low DOYmax (early maximum), while high values correlate with higher DOYmax (later maximum). Dashed lines indicate twice standard errors. Each short bar on the x-axis indicates an observation. A: End of snow melt, B: summed air temperature during the green-up period, C: summed rain during the green-up period, D: summed air temperature during the previous growing season. Estimated degrees of freedom are shown by each Y-axis.

DOYmax GAMM models: The timing of the maximum NDVI-FR is explained a lot better than SINDVI. The lowest adjusted R2 is 0.85 and the highest 0.98 indicating that the used explanatory variables are controlling most of the variance of DOYmax. The response curves for the combined vegetation types are shown in Figure 5. Changes towards later snow melt clearly indicates a later occurring maximum as does increasing air temperatures during the green-up period although the positive response disappear with very high green-up temperatures. Both rain in the green-up period and temperatures in the previous year show an optimum range for later timing although none of the responses are as pronounced as for ESM or AIRUP. The model explains 89% of the variance in the timing of the combined vegetation types.

A late snow melt and hence later onset of the growing season is significantly postponing the time of maximum in all vegetation types. The shape of the response for grassland indicates a slight negative response with very early snow melts but confidence intervals are so large here that this might be due to the relative small sample size. Higher temperatures and amount of light and rain during the green-up period respond in a later maximum for both the Cassiope heath and the Salix snow bed. The integrated vegetation greenness in the previous year (SINDVIPREV) is only included in a few of the models and in general do not seem to have a great impact on the timing of the greening in a following year.

## Discussion

### NDVI-FR measured in six vegetation types

As illustrated in Figure 3 and 4 we here document for the first time to our knowledge how NDVI-FR varies substantially between six vegetation types in the High Arctic. Other studies have reported on one or two vegetation types [e.g. [[15]] or from the lower arctic regions [e.g. [21]]. Not surprisingly, NDVI-FR shows lower max values in dry and sparsely vegetated areas than in moist and wet areas. For example, in 2005, NDVI-FR peaks around 0.43 in the fens whereas peak values for Dryas heath are around 0.35. Looking at plant species types, we found increasing values of MaxNDVI and SINDVI from moss and lichens at fell fields to evergreen and wintergreen species in Dryas heath and Cassiope heath and further to deciduous species in Salix heath, grassland and fen. Evergreen and wintergreen species invests energy in green leaves that are robust and can sustain the harsh winter conditions. Hence they develop small thick waxy leaves which are able to start photosynthesis right after snowmelt, or even before snowmelt [23]. These leaves require high amounts of energy to develop and sustain [24] and therefore less green tissue is exposed during the growing season than is the case for the deciduous plant types. This is mirrored in the NDVI-FR values (Figure 2). On the other hand, Salix arctica, fen species and grassland species have a high acute need of nutrients and water for development of green tissue during the growing season and hence deciduous plant species are found at moist and wet locations where water and nutrients are more abundant than in the drier ecotones.

Looking at DOYmax (Figure 3B) three different groups are evident. Fell field and Dryas heath peaks first followed by Cassiope heath, grassland and fen, whereas Salix heath peaks latest in the growing season. Fell field and Dryas heath are both situated at exposed plateaus where snow might blow off during winter months and which get snow free early in spring. Plant species here have an early snow melt and hence start of growing season (Figure 3D) and can take advantage of the high PAR levels around midsummer. Therefore they peak earlier than species growing where snow stays later. In the other end of the scale, Salix heath is often situated around late melting snow fans in the Zackenberg area [25], and hence, they experience the latest DOYmax. From Figure 3D it is evident that the growing season starts at approximately the same time at Cassiope heath, grassland and fen. These community types experience DOYmax at the same time (Figure 3B) and we conclude that this also is caused by the time of snowmelt.

### Timing

During the years of monitoring, we found a trend of earlier end of snow melt in all years after 2001 (Figure 3D), and hence, an earlier timing of MaxNDVI (Figure 3B) albeit this trend was only clear after year 2002. Therefore, we would expect increasing values of SINDVI. However, contrary to regional studies of greening in the Arctic [e.g. [26]] we found a decreasing trend of the NDVI-FR and SINDVI in some of the vegetation types at Zackenberg (Figure 3A). However, only a few of the trends are very significant (p < 0.01) and with the short monitoring period and known variability in the climate in NE-Greenland one year with opposite trend might reduce or remove the significance. There also is a tendency to trambling effects in the drier areas but given that the significance of decrease is highest in the wet areas we assume that trambling is currently not an issue. However, it seems that trambling is a factor that needs to be taken into account in the long time series analysis in the coming years. The decrease in NDVI-FR and SINDVI for some of the vegetation types combined with the simultaneous earlier end of snow melt (Figure 3D) this indicates that the availability of water from melting snow is important for the area. Therefore, although greening might occur wide spread in the Arctic there are variations on the local scale that will influence the regional trends in the longer term.

### The models

Monitoring growth dynamics of the vegetation types in the High Arctic with remote sensing is influenced by a range of variables (clouds, surface moisture, access etc.). The need to be able to model the growth dynamics from the reliable measurements is therefore clear. Many different models describing the evolution of NDVI-FR through the growing season has been published. Fisher introduced a double-logistic function for modelling of growing season by NDVI in farm land [27] while Lüdeke et al. used a three-spline function in the global Frankfurt Biosphere Model [28]. An intersecting straight lines model was used by Shibayama et al. for detecting phenophases in the Subarctic [29]. The maximum composite technique of filtering AVHRR-based NDVI values and then use of the data directly with linear interpolation have been applied world-wide including in the subarctic Alaska [21]. However, the latter method need data regularly from the growing season which is not possible in the high arctic NE-Greenland where field researchers depending on the year arrives after the start of the growing season. Several of the mentioned methods have been accessed for this dataset but fits were not as good as those obtained with the 2nd degree model. We therefore conclude that for the relative sparse high arctic vegetation a quadratic model is the best for simple and quick estimation of growth dynamics. Marchand et al also used this for high arctic vegetation [15]. The model can be easily applied and works for separate vegetation types in the High Arctic as for the vegetation as a whole.

The response variables (SINDVI and DOYmax) describing the integrated vegetation greenness and timing of maximum was modelled successfully. Unfortunately, MaxNDVI was not possible to model. This is probably based on two reasons: 1) very little variance in MaxNDVI from year to year and 2) uncertainties in measurements of +/- 0.02 due to surface moisture, reflection or shadowing of the surroundings (field investigator, boulders etc) and the view angle of the instrument at the time of measurement. For example, Jacobsen and Hansen found a clear relation between soil moisture and MaxNDVI in Zackenberg [30]. However, changes in MaxNDVI between vegetation types are still clear (Figure 2 and Figure 3) and can be used for future monitoring of changes in vegetation composition.

The mixed models used in this study are complex models that are not easily interpreted – especially if many degrees of smoothing have been used. We have limited the amount to three degrees of smoothing resulting in biological meaningful explanation of the controlling environmental factors. Fewer degrees would restrict to almost linear relations in most cases and more degrees will cause the model to follow the variance and not the overall pattern of the relationships. Unfortunately, it is not possible to obtain either the variance explained by each variable with the given method nor is it possible to interpret variables importance between models. Though it has not been possible to estimate the variance explained by each variable we believe SINDVI in the previous year to be the most important. Yet, the remaining variables in the models are also significant and therefore explain some of the variance although the level explained is uncertain. Other simpler models have been presented [e.g. [21]] but these have not produced satisfactory explanation.

### Explanatory variables

Table 2 shows the explanatory variables to the models of SINDVI and DOYmax. It is outstanding that we here document that SINDVI and DOYmax are differently determined by the explanatory variables in different vegetation types. We expected to see that dry, moist and wet vegetation types showed similar trends of explanatory variables in the sense that they would be similar within either dry, wet or moist vegetation types and differ markedly between them. However, this is not the case and other explanations must be found, since no patterns seem obvious. We speculate that the geographical distribution pattern of the individual dominant species within each vegetation type may be more dominant for the direction of each explanatory variable within the individual vegetation types than whether the vegetation type is situated on dry, moist or wet ground. In the following we analyze and discuss those variables which seems most biological meaningful.

SINDVI: In general, the R2 values for the models of SINDVI are satisfactory except for the model of Dryas heath where R2 only reaches 0.21, and other factors must explain SINDVI. Looking at all models, two variables (temperature the previous year and SINDVI the previous year) show the same directional output for both the model of SINDVI for all vegetation types together and the individual models for each vegetation type (Table 2). Temperature in the previous year shows a negative relationship with SINDVI (Figure 4F), and therefore, higher temperatures during the growing season causes lower SINDVI the following year. The reason for this could be that high temperatures have been shown to increase the number of flowers in the following year [14]. This would result in lower NDVI-FR simply because of the brighter colours of the flowers and lower absorption by chlorophyll but also in less greenness since the photosynthates are used for setting flowers and not for biomass growth. Another reason could be the effect of increased temperatures where snow patches disappear early in the season and the soil dries out causing low soil moisture levels. This would limit growth and build-up of resources for the following year [31]. Contrary to this, SINDVI the previous year shows a positive correlation with SINDVI the current year except in grassland where no relationship exists (Table 2, Figure 4E). Almost all high arctic plant species are perennials and store resources in storage organs (leaves, stems, roots, rhizomes) from year to year [32-34]. Assuming that high values of SINDVI indicates good growth conditions the previous year, the plants probably have build up resources in storage organs for growth the current year, and this is what is mirrored in high SINDVI values. A third variable, temperature during the growing season (AIRGS) seems interesting. For the overall model and for the Cassiope heath, Salix heath and grassland models a non-linear response with a maximum for SINDVI is shown (Table 2, Figure 4B). That is, SINDVI increases with increasing temperatures to a certain point where after SINDVI decreases as temperatures get higher. The explanation to this could be that the dominant species of these vegetation types all have a northern distribution pattern in Greenland and are not common at lower latitudes [35]. Hence, they grow close to a regional southern distribution limit, and only a temperature increase to a certain degree increases their photosynthesis. Larger increases in temperature limit the photosynthesis, ceasing growth and causing SINDVI to decrease [36-38] probably due to enzymatic inhibition of the photosynthetic apparatus. Since temperatures peaks in late July and early August we do not expect the negative response from higher temperatures to be an effect of day length.

DOYmax: In general a higher variance can be explained by the models of DOYmax than SINDVI for all vegetation types (Table 2), but patterns in the individual explanatory variables for the six vegetation types are difficult to locate except for time of snowmelt. All vegetation types except grassland shows a positive correlation between this variable and DOYmax meaning that the later the snow melts, and hence the start of the growing season, the later the DOYmax is reached. This seems logic since all plant species needs a certain length of time after snowmelt to assimilate carbon and reach maximum biomass. This is also obvious from Figure 3B and Figure 3D where DOYmax appears in three different groups depending on time of snow melt. Other factors than time may however, influence this period, for example air temperature. Our model shows a positive correlation between temperature and DOYmax in all vegetation types except grassland and fen meaning that high temperatures in the green-up period delays DOYmax. Our only explanation to this phenomenon is as described above that too high temperatures may inhibit photosynthesis in the species growing close to their regional southern limit or increase flowering that will lower the build-up of green plant material. In conclusion we believe that the high R2 values for all vegetation types for the modelled DOYmax is due to a strong correlation with time of snow melt.

### Climatic change in the Zackenberg area

Climatic changes in the Zackenberg area is currently believed to show mainly as increased temperatures and winter precipitation [39]. Increased precipitation will result in more snow hence prolonging the snow cover. This would lead towards decreasing greenness and later timing of the maximum. However, Hinkler investigated the snow distribution during future climate scenarios and concluded that the projected increased precipitation and higher temperatures will leave summer-snow cover depletion close to status quo [40]. Recent years (e.g. 2002) have shown examples of this where a deep snow cover quickly can melt away if the temperatures in the spring are higher than normal. In the case of year 2002; it had the next highest recorded snow depth (50% above normal) at the end of winter but the snow melted off only one day later than the average melt off [41].

Further, the unclear response from the high arctic locations of Walker et al. [13] combined with the decrease in SINDVI from 1999 to 2005 in our study enhances the need for further studies in these areas. Also, the clear difference in the controlling variables and their induced response in the growth dynamics between vegetation types highlight the importance of high resolution studies. In regional climate change models the growth dynamics are often supplied by AVHRR and satellites with similar resolution. A regional study based on recent higher resolution satellite data (ASTER, SPOT etc.) and based on the methods presented here would supply valuable new knowledge in this area – an area that clearly needs attention for future regional or global climate modelling.

## Conclusion

Although greening is reported to occur widespread in the Arctic, we have shown that variations exist that might influence regional trends in the longer term. We investigated the growth dynamics for six dry, mesic and wet vegetation types using NDVI-FR and generalized additive mixed models (GAMM). We found a quadratic model to be the best for simple and quick estimation of seasonal integrated NDVI-FR and used this in the analysis. Snow melt and temperature were of major importance for the timing of the maximum growth as well as for the seasonal growth. More than 85% of the variance in timing of the maximum growth is explained by the models and similar for the seasonal growth of mesic and wet vegetation types. We found several non-linear growth responses to the environmental variables and conclude that the uses of GAMMs are valuable for investigating growth dynamics in the Arctic.

## Methods

### Field site and vegetation types

The study area was located in the high arctic Zackenberg valley in Northeast Greenland (74°30'N, 21°0'W) (Figure 1). The climate of the area is high arctic with an annual mean temperature ranging between -8.5°C to -10.1°C (1996–2005). Mean monthly temperatures only rise above freezing during June, July and August and occasionally September with a mean temperature during the 1995 to 2005 period of 1.9°C, 5.7°C, 5.0°C and -0.9°C, respectively. Minimum July temperature during the 1995 to 2005 period was -2.6°C and maximum was 21.8°C. Precipitation typically falls as snow and only around 15% falls as rain. The annual precipitation was 261 mm water equivalent (1996–2005) with a minimum of 128 mm in 2002/2003 and a maximum of 416 mm in 1998/1999. Time of snow melt depends on several factors (e.g. topography, precipitation, temperatures) but in general varies between late May and mid June for most vegetation types. Details and climate data can be found in the yearly report for Zackenberg Ecological Research Operations or through the database .

The vegetation in the valley consist of sparsely vegetated areas (abrasion plateaus, fell fields), dry and mesic heaths (Dryas spp., Cassiope tetragona heath and Vaccinium uliginosum heath), snowbeds (Salix arctica) and wet grasslands and fens dominated by Arctagrostis latifolia, Eriophorum triste and Eriophorum scheuchzeri, Dupontia psilosantha, respectively [25]. The vegetation is relatively homogenous with areas of Cassiope heath or Dupontia fen covering large hectare sized patches. A typical progression of vegetation types occurs along hillside and moisture gradients. Dry abrasion plateau with fell field and open Dryas heath is located on the top of small hills. Moving from the hill top down slope towards a river bed or other depression the Cassiope heath takes over followed by Salix arctica snowbed which is covered by snow drifts in the first part of the growing season. Near the bottom the grassland is located beneath the snow drift and fens follow where running water is available through most of the growing season.

### Field measurements

**Figure 6:**
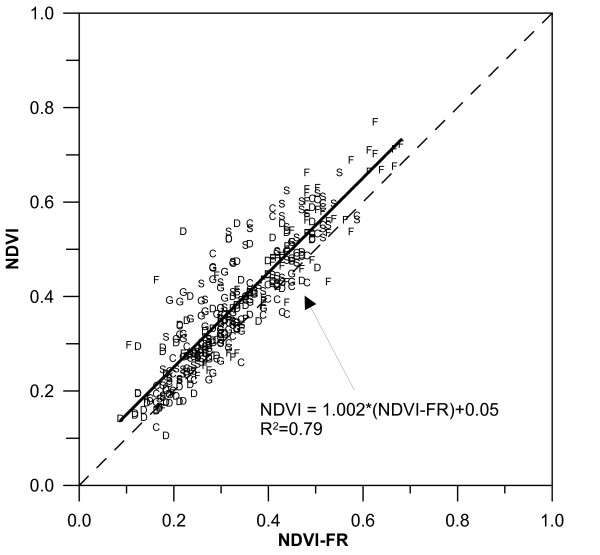
**Relationship between NDVI-FR and NDVI**. Each data point is marked by a letter indicating the vegetation type for that plot measurement. C: *Cassiope *heath, D: *Dryas *heath, F: Fen, G: Grassland, S: *Salix *heath. The linear relation is shown in solid (R2 = 0.79, n = 390, p < 0.0001) and the 1:1 line in dashed.

All data used in this study originated from the BioBasis and ClimateBasis monitoring programs under Zackenberg Ecological Research Operations (ZERO). Field measurements were done in 26 plots scattered around in the valley (Figure 1) covering the six major vegetation types: Fell field, *Dryas *heath, *Cassiope *heath, *Salix *heath, grassland and fen. All plots were closer than 1 km from the central climate station. This study included 22 plant plots from the BioBasis program where NDVI-FR is measured regularly. At Dry1, Sil1, Sil2 and Sal1, the snow melted so early that the time of snow melt was not possible to estimate. The plots varied in size from 2 m^2 ^to 300 m^2 ^and were not all homogenously vegetated. They differed in size because the plots were originally intended for flowering studies of species monitored under most of the occurring habitat conditions (biotic and abiotic), with a possibility of counting 50 or more flowers at each location. NDVI-FR measurements were done in each corner of 4 or 8 sub areas in each plot depending on the setup of the plot [42]. Measurements were performed once every week during the field season (1 June to 31 August) from 1999 to 2005. A Skye SKR110 instrument  with narrow band filters centred at 660 nm and 730 nm were used for the measurements. The field of view for the instrument was approximately 3 m^2 ^when used at a height of 1 m. Each measurement was carried out at the same position with nadir viewing. The instrument was calibrated every second year to avoid drift. Standard NDVI use measurements with a band centre around 900 nm instead of 730 nm. Hence, this paper therefore uses NDVI-FR instead of NDVI. Measurements in 2004 of both NDVI-FR (Skye sensor) and NDVI (ASD inc. Fieldspec Hand-held radiometer) showed a 79% correlation between the datasets (R2 = 0.79, n = 390, p < 0.0001) (Figure 6). Hence, 21% of the variance cannot be explained by the other parameter. This is due to the use of different instruments and especially the location of the far-red (FR) band. FR is situated on the red-edge slope. Any changes in NDVI-FR compared to NDVI other than from changes in absorption by pigments will therefore be due to changes in the position of the red-edge slope. Senescence and water stress have been shown to cause a blue shift of the red edge [43] resulting in a higher FR and hence a NDVI-FR increase. Near-infrared (NIR) bands on the other hand will experience a decrease during senescence leading to opposite trends in the two indices although depending also on the slope of the red-edge and absorption in the red bands. However, the two indices are closely related and results from this NDVI-FR study are therefore also valuable for comparison with similar NDVI studies.

In order to keep the nomenclature simple we have used NDVI in the explanatory variables names (e.g. MaxNDVI). The resulting number of observations is shown in Table 1. However, this study looked at the vegetation response and not species response and therefore needed to counter the heterogeneous nature of the plots. Hence, each of the sub areas in the plots was classified into one of the six vegetation types based on cover estimates of the major shrubs (*Cassiopa tetragona*, *Dryas *spp., *Vaccinium uliginosum*, *Salix arctica*), herbs, graminoids, mosses, lichens and bare ground. The vegetation types covered dry, mesic and wet ecosystems and were similar to those defined by Bay [25]. These six types cover more than 70% of the valley and hence, are representative for the area. Including more vegetation types would be difficult since the limited range and dynamics of NDVI-FR and size of vegetation patches within the High Arctic are limiting factors for the number of vegetation types that can be explored [44]. It was assumed that the vegetation types for each sub area did not change significantly during the period of analysis (1999–2005).

All standard climate variables (temperature, wind, humidity, radiation, etc.) were measured year-round at the climate station that was placed in the middle of the study area (Figure 1).

### Snow melt and NDVI-FR seasonal modelling

**Figure 7:**
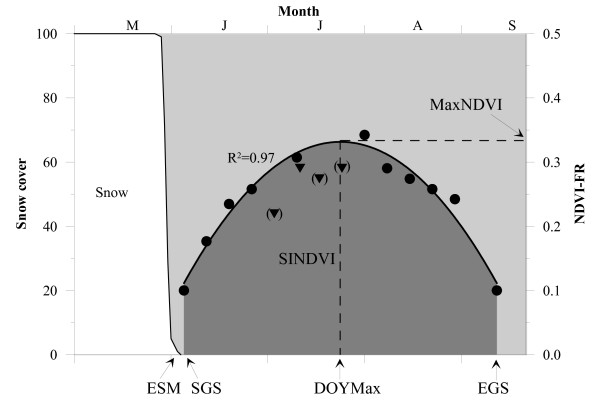
**Growing season and main response variables defined by snow melt and NDVI-FR**. ESM: End of Snow Melt, SGS: Start of Growing Season, DOYmax: Day of maximum NDVI-FR, EGS: End of Growing Season, SINDVI: Seasonal integrated NDVI-FR. Brackets indicate measurements that have been removed (see text for further details).

An example of field data from the NDVI-FR plots and how the main response variables were extracted is shown in Figure 7. Percent snow cover was monitored at each plot until all snow was gone. The last day where snow was monitored was defined as End of Snow Melt (ESM). Since the actual day where all snow had melted was not known and that plants may utilise radiation and start growth under very shallow snow packs [23], the day after last observed snow cover (ESM + 1) was defined as the Start of Growing Season (SGS). Plots were only monitored once a week and to avoid different bias between plots we used the described method. For plots where no snow was present in the beginning of the field season (14 out of 141 plots) ESM was estimated by linear regression with ESM from plots that had snow. The correlations where all significant (p < 0.05) with R2 values higher than 0.9. For one plot (Sil3) there were to few points to do it directly from DOY with significant relation and summed temperatures (base: 0°C) was used instead with a significant result and a R2 of 0.82.

The vegetation reaches maximum in mid summer after which the greenness fades towards the end of the growing season (EGS), which was defined as the day soil near the climate station at 2.5 cm depth reached freezing for two subsequent days. SGS and EGS were set to a NDVI-FR-value of 0.1 representing the vegetation before and after active growth. A NDVI value of around 0.1 is typical for arctic studies [26,45]. The period from the start of growing season to the maximum was defined as the green-up period. In order to calculate the exact date (DOYmax) and level (MaxNDVI) of maximum NDVI-FR a quadratic model was fitted to the NDVI-FR measurements. This model was also used to calculate the seasonal integrated NDVI-FR (SINDVI) from ESM to EGS. The quadratic model has previously been used in the High Arctic [15,20]. Other methods are discussed below.

Some of the original NDVI-FR data were unreasonable low. This could be caused by several factors where flowering, thin clouds, shadow or dew are the main factors [e.g. [46,47]] and these data were removed before fitting the quadratic model. Most of the removed measurements occurred in the green-up period with high numbers of flowers. The removal procedure went through each data series for each season and checked that all values from SGS to DOYmax were growing. If a value was lower than the previous and DOYmax had not been reached it was removed. The same procedure was done from EGS to DOYmax. An example of removed data can be seen in Figure 7.

### Explanatory variables

The explanatory variables used in the modelling were primarily obtained from the climate station and included:

1) the time for end of snow melt (ESM), 2) the summed air temperature (base of 0°C [45]) through either the entire growing season (AIRGS) or during the green-up period (AIRUP), 3) the summed incoming PAR through either the entire growing season (PARGS) or during the green-up period (PARUP), 4) the summed rain through either the entire growing season (RAINGS) or during the green-up period (RAINUP), 5) the seasonal integrated NDVI-FR in the previous year (SINDVIPREV) and 6) the summed temperature (base of 0°C) through the previous growing season (AIRGSPREV). PAR was not measured at the climate station until 2003 and we therefore estimated it from shortwave incoming radiation for 2000, 2001 and 2002 using linear regression (p < 0.001, R2 = 0.99).

### Trend analyses

We performed trend analyses on the response and explanatory variables using the seasonal Mann-Kendall method [48]. This method is widely used in environmental science, because it is simple, robust and can cope with missing values [e.g. [20]]. We used the software developed by Libiseller [49].

### Modelling framework

We modelledeach response(e.g.percent coverageof *Dryas*, *Salix heath*, *Cassiope*, fen, and grass) as a function of the following fixed effects:end of snowmelt, summed air temperature, summed PAR, summed precipitation, summed temperatures andseasonally integrated NDVI (SINDVI) in the previous year, with samples asrandom effects nested withinblocks identified bysite id. For SINDVI the explanatory variables were taken for the entire growing season while in the modelling of the timing and level of maximum we only used the data from the explanatory variables during the green-up period.

The initial models for the three response variables were:

SINDVI = s(ESM) + s(AIRGS) + s(PARGS) + s(RAINGS) + s(SINDVIPREV) + s(AIRGSPREV)

DOYmax = s(ESM) + s(AIRUP) + s(PARUP) + s(RAINUP) + s(SINDVIPREV) + s(AIRGSPREV)

MaxNDVI = s(ESM) + s(AIRUP) + s(PARUP) + s(RAINUP) + s(SINDVIPREV) + s(AIRGSPREV)

where s() is a function with smoothing of up to 3 degrees of freedom.

We performed the modelling in the open-source statistical software R (vers. 2.3.1.) using a generalized additive mixed model (GAMM). The mixed model is particularly useful in that it allows for the spatial pseudo-replication that occurs when using each of the four to eight measurements in the plots as separate measurements [50]. The GAMM also allows for the possibility of non-linear relationships and is available under the mgcv (multiple smoothing parameter estimation by generalized cross validation) library in R and uses the gaussian family as a default [51]. Three degrees of freedom (smoothing) was used. For each of the 21 models all specified variables where used in the first run. If all variables were significant no further steps were taken. If one or more variables were not significant (p > 0.05) the variable with the lowest significance was excluded and the model rerun. The Akaike's Information Criteria (AIC) [52] and the ANOVA model p-value where used to evaluate if the new model performed better – the lower the AIC, the better the model. In the case that there was no significant difference between the models (p > 0.05) and only very little difference between the AIC the simplest model was chosen. This procedure continued until only significant variables were included. If no convergence was met with all variables, PAR was excluded and the model run without it. Then the above procedure was followed until the best model was found. If no convergence persisted the model was not successful. A summary with significance for each explanatory variable and adjusted R2 were produced for each final model as were response curves showing the relationship between each variable and the response variable for the given model. Each response curve was the result of backfitting the GAMM model to calculate the additive contribution of each variable using non parametric smoothing. Thus, they can be interpreted as a transformation of the response variable (e.g. SINDVI) so that low values on the y-axis correlate with low SINDVI (or DOYmax or MaxNDVI), while high values correlate with higher SINDVI.

## Competing interest

The author(s) declares that there are no competing interests.

## Authors' contributions

MPT initiated the study, carried out the analyses and wrote the manuscript. LI contributed throughout the manuscript writing especially on the botanical parts. BUH supported in drafting the study and contributed to the writing. MSW supplied the GAMM programming and contributed to the statistical parts. All authors read and approved the final manuscript.
